# A common metric for questionnaires on health anxiety in cancer patients

**DOI:** 10.3389/fpsyg.2024.1455121

**Published:** 2024-12-03

**Authors:** Michael Friedrich, Thomas Schulte, Merle Malburg, Andreas Hinz

**Affiliations:** ^1^Department of Medical Psychology and Medical Sociology, Leipzig University, Leipzig, Germany; ^2^Comprehensive Cancer Center Central Germany (CCCG), Leipzig, Germany; ^3^Rehabilitation Clinic Bad Oexen, Bad Oeynhausen, Germany

**Keywords:** health anxiety, cancer, worry, fear, progression, recurrence, common metric, conversion

## Abstract

After a cancer diagnosis, life is accompanied by worries, concerns and fears that the disease will recur or worsen. These can be normal and useful reactions but may also become so severe that they interfere with everyday functioning. A challenge for patient care is that the theoretical similarity of these reactions, is not consistently conceptualized in practice, hence the empirical comparability of their measures is unclear. Here we intend to show that the theoretical similarity is also empirically justified, and we present a common metric in graphical form that allows direct comparisons between different questionnaires. A total of 1,733 cancer patients completed the Fear of Progression Questionnaire Short Form, Questionnaire on Stress in Cancer Patients–revised version, Concerns About Recurrence Questionnaire, the subscales Health Distress and Negative Health Outlook of the EORTC QLQ-SURV100, and the Whiteley Index. Using a model based on item response theory, we linked the score values of the individual questionnaires. The main outcome of this study is a diagram that can be used to convert the respective values of eight questionnaires on health anxiety to another. All instruments showed a reliability above 0.75 near the mean health anxiety level. The common metric can be used to compare measurements with these questionnaires in terms of the level of health anxiety. Additionally, the reliability of the instruments can be judged at different levels of anxiety. This allows for a better comparability of test results and facilitates communication about the results among experts and with patients.

## 1 Introduction

Between 2010 and 2019, the number of new cancer cases increased by more than 25% worldwide (Kocarnik et al., 2022), while the global population only increased by around 11% in the same period (OECD, 2024). With approximately 10 million cancer deaths in 2019, the number of years in good health lost to cancer was estimated at 250 million years. This puts cancer in second place after cardiovascular diseases in the global comparison of causes of death and lost years of healthy life (Kocarnik et al., 2022).

Life with a cancer diagnosis is often accompanied by worries and concerns and fears, including fear of cancer recurrence (FCR), the fear of symptoms getting worse (fear of progression, FOP), and concerns about associated negative effects on the future. These are normal reactions to living with such a disease, but they can also become dysfunctional, affecting quality of life and daily functioning.

From a broader perspective, these worries, concerns and fears can be summarized under the concept of health anxiety (HA), a term that is also applied to chronic diseases. However, the fact that these feelings can be subsumed under one term does not make the questionnaires interchangeable. Instead, there may be different or even contradictory interpretations of the same phenomenon (Maheu et al., 2021). So far, there is a lack of empirical evidence as to whether interchangeability is justified and, if so, in what way. This may be one of the reasons why there is still no consensus on where to draw the line between functional and dysfunctional anxiety, i.e., at what point HA should be considered clinically relevant.

For cancer, prevalence rates of at least moderate FCR vary between 22 and 87%, which is attributed to the use of different measurement tools (Luigjes-Huizer et al., 2022). Given this background, the prevalence rates of dysfunctional HA for different chronic diseases are estimated to be at least 20% (Lebel et al., 2020).

In summary, there are two main obstacles to planning patient care based on the results of different questionnaires, which should theoretically be comparable: the lack of clarity about the interchangeability of different perspectives and the use of different measurement tools.

The aim of this study is to overcome these obstacles and to relate scores on certain instruments to those on others, using a common metric. This would enable a comparison of the different perspectives in terms of their common quality.

Common metrics combine the total values of different instruments based on a commonality inherent in all instruments in order to convert the score from one instrument into the respective score of another or into standardized T-scores of larger item banks, e.g., a Patient-Reported Outcomes Measurement Information system (DeWalt et al., 2007). They are usually presented in tabular or graphical form or, alternatively, as a web application (Fischer and Rose, 2016). More information about common metrics in general and their underlying methods can be found, for instance, on https://www.prosettastone.org or https://www.healthmeasures.net. An overview of item banks for pain, fatigue, negative affect, physical function, and social function provided support for their validity across varied clinical populations with chronic conditions (Cook et al., 2016). Another study demonstrated the validity of US-based item banks for depression and anxiety in an independent sample of Australian adults (Sunderland et al., 2018).

In addition, there are further common metrics for psychological distress (Batterham et al., 2018), personality disorder (Zimmermann et al., 2020), and sleep quality (Friedrich et al., 2024), but not for HA.

The development of a common metric for HA was guided by the following three questions: (1) Do the instruments cover a common quality that justifies interchangeability (dimensionality of health anxiety)? (2) How reliably do the instruments measure the common quality and how accurate are the links between the instruments (measurement precision)? (3) How can measured values of one instrument be converted into values of another instrument (common metric)?

## 2 Materials and methods

### 2.1 Sample of cancer patients

From July 2022 to June 2023, patients at an oncology rehabilitation clinic were asked to take part in the study. During their stay, which usually lasts 3 weeks, patients receive physical fitness exercises, physiotherapy, training in relaxation techniques, and counseling on occupational and nutritional issues. Patients were consecutively included if they met the following criteria: Age of at least 18 years, confirmed cancer diagnosis, sufficient knowledge of German and no severe cognitive impairment. Out of a total of 2,250 surveyed patients, 1,733 (77%) met the inclusion criteria and gave their informed consent to participate. The study was approved by the Ethics Committee of the Medical Faculty of the University of Leipzig (approval number: 513/21-ek).

### 2.2 Instruments

#### 2.2.1 Fear of progression (FOP-Q-SF, FOP-Q-RS)

The Fear of Progression Questionnaire Short Form, FoP-Q-SF, (Mehnert et al., 2006; Mehnert et al., 2009) is a short form of the 43-item Fear of Progression Questionnaire (FoP-Q) (Herschbach et al., 2005). The 12 items are to be answered on a five-point Likert scale (range 1–5), with a total score between 12 and 60. Scores of 34 and above are considered indicators of dysfunctional FOP (Hinz et al., 2015; Herschbach et al., 2010).

An even shorter version (Fear of Progression Rapid Screener, FOP-Q-RS) was developed from the short form, which only contains 5 of the 12 items, and has a score ranging from 5 to 25. Values of 12 and above are associated with a moderate level of generalized anxiety disorder (Youssef et al., 2021).

#### 2.2.2 Stress in cancer patients (QSC-R23-ANX)

The Questionnaire on Stress in Cancer Patients – revised version, QSC-R23, (Herschbach et al., 2003) contains 23 items that represent five scales: psychosomatic complaints, anxiety/fears, information deficits, everyday life restrictions, and social strains. In this study, we limited ourselves to the anxiety subscale, QSC-R23-ANX, as its four items relate directly to the fear of cancer progression and to effects on the future. The items have a value range from 0 to 5, the score also ranges from 0 to 5, and higher values mean greater anxiety.

#### 2.2.3 Concerns about recurrence (CARQ-4, CARQ-3)

The Concerns About Recurrence Questionnaire (CARQ-4) (Thewes et al., 2015) comprises four questions to assess the fear of cancer recurrence. The first three items are measured on an 11-point Likert scale from 0 to 10. The fourth item asks about the subjective probability (range 0–100) of cancer recurring; its value range is also transformed to the range 0–10. High values represent a greater fear of recurrence of the cancer. The total score of all four items (CARQ-4) has a value range from 0 to 40, with values of ≥12 indicating clinical levels of FCR.

A second questionnaire, the CARQ-3, refers only to the first three of the above-mentioned items. Its total score ranges from 0 to 30, with values of 10 and above indicating clinical levels of FCR (Thewes et al., 2015). Two independent psycho-oncological research teams translated the questionnaire from English into German. The questions in the original version, which were specifically tailored to breast cancer patients, were adapted to ensure that they can be answered by cancer patients in general. Differences between the two versions were reconciled by consensus under the guidance of a clinician with relevant practical experience in conducting patient surveys.

#### 2.2.4 Worries of cancer survivors (SURV-HD, SURV-NHO)

The EORTC QLQ-SURV100 (van Leeuwen et al., 2023) is a new questionnaire designed to measure all aspects of health-related quality of life that are important to cancer survivors. The questionnaire contains several symptom scales, two of which have been included in this metric: Health Distress (SURV-HD) with three items regarding worries about health and cancer recurrence, and Negative Health Outlook (SURV-NHO) with seven items about future aspects of health. The items are to be answered on a four-point scale (range 0–3). Each scale is linearly transformed into the range 0–100, and higher values indicate a higher level of worry about health.

#### 2.2.5 Illness worries (Whiteley-7)

The Whiteley Index, Whiteley-7 (Fink et al., 1999), is a shortened version of the original 14-item binary-coded Whiteley Index (Pilowsky, 1967) as a screening instrument for somatization illness. The response format of the seven items used here is five-point Likert-scaled (0–4), the sum score range is between 0 and 28 (Carstensen et al., 2020), with higher values indicating greater worry about illness. The questionnaire in its current form contains an additional item on obsessive illness rumination, that is used to evaluate two different score versions. In the presentation of the conversion between the instruments, however, we will confine ourselves to the originally validated version with seven items.

Altogether 38 items were included in the model on which the conversion between questionnaires is based (number of items for FOP: 12, QSC: 4, CARQ: 4, SURV: 10, Whiteley: 8).

The metric formed by the items of the five instruments contains all four dimensions of HA (Longley et al., 2005), even if not all dimensions are always included in the individual instruments. Thus, the following dimensions of HA are covered by the respective instruments: FOP: affective, cognitive, perceptual; CARQ: affective, cognitive; SURV: affective, cognitive, behavioral, perceptual; Whiteley: affective, cognitive, perceptual.

### 2.3 Statistical analysis

The instruments were linked together in a single group design: they were collected in one survey and could thus be calibrated concurrently. The model parameters were estimated using item response theory (IRT) on the basis of the graded response model. To ensure that the two basic assumptions of IRT (local independence and appropriate dimensionality) (Embretson and Reise, 2000) were met, we developed a bifactor model (Reise et al., 2010; Reise, 2012) with one general factor for HA and one additional specific factor. The latter captures the items with an occupational reference (e.g., fears or anxiety regarding occupational performance) and thus separates external influences on the HA that would otherwise have led to residual correlations between items that cannot be explained by the common factor (local dependence).

The degree of local (in)dependence was assessed by evaluating the residual correlation matrix. According to simulations, it can be assumed that independent items are unlikely to show residual correlations of more than 0.3 above or below the average residual correlation (Christensen et al., 2017).

Three coefficients are relevant to judge the appropriateness of dimensionality: The first measure is the proportion of score variance that can be attributed to the general factor (coefficient omega hierarchical, COH). If COH is greater than 0.8, it can be assumed that the general factor is the dominant source of systematic variance, that is, the common score is assumed to be essentially unidimensional (Rodriguez et al., 2016).

The second measure is the proportion of common item variance that can be attributed to the general factor (explained common variance, ECV). Values greater than 0.7 indicate a strong general factor, that is, its loadings are not substantially biased by other sources of systematic variance (Rodriguez et al., 2016).

The third measure comprises the percentage of correlations that are not contaminated by multidimensionality (percentage of uncontaminated correlations, PUC). With values greater than 0.7 (Rodriguez et al., 2016), the common variance can be considered sufficiently unidimensional. ECV and PUC should be interpreted together, as the relevance of ECV in the assessment of dimensionality decreases with increasing values of PUC.

Measurement precision refers to the accuracy with which individual scores can be used to indicate the level of HA. It is determined by the standard error of measurement (SEm, to avoid confusion between this abbreviation and the usual abbreviation for structural equation modeling, SEM, we use the lowercase letter “m” here), which is directly related to reliability via the formula Reliability = 1 – (SEm/SD)^2^. A distinguishing feature of IRT compared to classical test theory (CTT) is that the reliability can be determined for each individual score value. Unlike in CTT where the SEm is a constant, in IRT it is variable, as it depends on the test information function.

The accuracy of the links between the instruments will be assessed on the basis of the mean of the differences between the measurements of two instruments (methods): When measuring HA by two different methods, the measurements usually show a certain disagreement (lack of agreement), which can be summarized by the mean of the differences of method A minus method B. The 95% confidence interval of this mean indicates the magnitude of the systematic difference (Giavarina, 2015), i.e., systematic over- or underestimation of method B compared to method A.

Additionally, we present the correlations between the raw scores of the measures and their respective estimated theta values (Supplementary Table 1) and Bland–Altman plots (Bland and Altman, 1995, 1999; Giavarina, 2015) to show the agreement among the estimated theta values on the common metric (Supplementary Figure S1).

The analyses were conducted with R, version 4.3.2 (R Core Team, 2023) using the packages mirt, version 1.4.1 (Chalmers, 2012) for the IRT-based analyses, and ggplot2, version 3.4.4 (Wickham, 2016) to create the graphs.

## 3 Results

### 3.1 Sample characteristics

Table 1 presents the characteristics of the sample. Out of 1,733 participating patients, 59.5% were female, the mean age was 56.1 years (SD = 14.5 years), and 37.8% were retired or unemployed. The most frequent diagnoses were tumors of the breast (32.3%), the prostate (17.8%), and tumors of the gastrointestinal tract (16.7%).

**Table 1:** Sociodemographic and clinical characteristics of the sample (*n* = 1,733).

	*n*	(%)
Sex		
Male	702	(40.5)
Female	1,031	(59.5)
Age group		
18–39 years	254	(14.7)
40–49 years	276	(15.9)
50–59 years	417	(24.1)
60–69 years	464	(26.8)
≥70 years	322	(18.6)
Education^a^		
Elementary school (8–9 years)	356	(20.6)
Junior high school (10 years)	527	(30.5)
High school/university (≥11 years)	830	(48.1)
No formal qualification	13	(0.8)
Employment status^a^		
Employed	996	(57.7)
Unemployed	63	(3.7)
Retired	589	(34.1)
Other	78	(4.5)
Tumor localization		
Breast	560	(32.3)
Prostate	309	(17.8)
Gastrointestinal tract	290	(16.7)
Hematological	202	(11.7)
Female genital organs	108	(6.2)
Urinary tract	87	(5.0)
Melanoma	49	(2.8)
Thyroid/endocrine glands	38	(2.2)
Male genital organs	29	(1.7)
Others	61	(3.5)
Treatment		
Surgery^a^		
No	177	(10.2)
Yes	1,556	(89.8)
Radio therapy^a^		
No	952	(55.0)
Yes	779	(45.0)
Chemotherapy^a^		
No	882	(51.1)
Yes	843	(48.9)
Hormone therapy^a^		
No	1,247	(72.5)
Yes	473	(27.5)
Antibody therapy^a^		
No	1,452	(84.7)
Yes	262	(15.3)

^aMissing data not reported.^

### 3.2 Dimensionality of health anxiety

Both basic assumptions of IRT held after assuming a general factor and a specific factor (systematic influence) regarding three items with occupational reference. Residual correlations showed values from −0.15 to 0.26, the mean of residual correlations was 0.01. Hence, all correlations were within the interval of 0.01 ± 0.30, local independence was assumed.

The score variance attributable to the general factor (COH) was 0.97, thus the dominant influence on the score is a common (general) factor. Furthermore, the item’s variances explained by the general factor were not biased by the assumption of the specific factor, because ECV was 0.91. More than 99% of the correlations between the items were not contaminated by specific factors (PUC > 0.99).

### 3.3 Measurement precision

**Figure 1:**
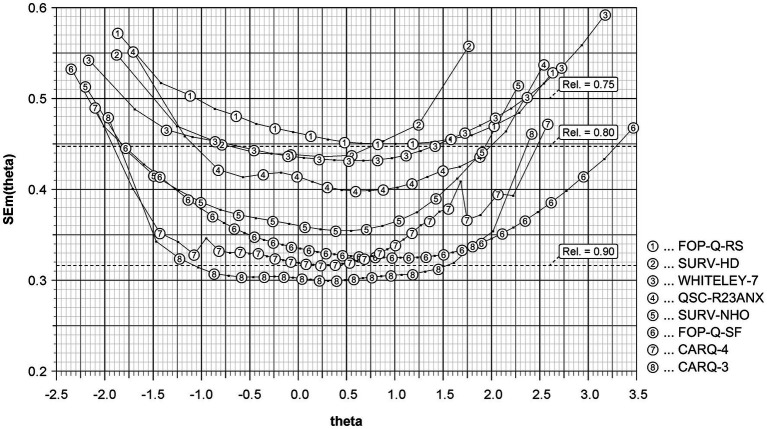
Measurement precision of the instruments to measure health anxiety. FOP-Q-SF, Fear of Progression Questionnaire Short Form; FOP-Q-RS, Fear of Progression Rapid Screener; QSC-R23-ANX, anxiety subscale of the Questionnaire on Stress in Cancer Patients – revised version; CARQ-4, 4-item version of the Concerns About Recurrence Questionnaire; CARQ-3, 3-item version of this questionnaire; SURV-HD, subscale Health Distress of the EORTC QLQ-SURV100; SURV-NHO, subscale Negative Health Outlook of this questionnaire; WHITELEY-7, Whiteley Index; theta: standardized factor score (mean = 0, standard deviation = 1); SEm(theta), standard error of measurement.

The accuracy with which the level of HA can be inferred from individual score values can be seen in Figure 1. It shows the SEm of the general factor in dependence from the standardized factor score (theta).

Each data point corresponds to a possible sum value of the respective instrument and has a number that identifies the instrument it belongs to. All instruments achieved a reliability of more than 0.75 within a theta-range of ±1 standard deviation (SD). In this range the CARQ-3 showed the best reliability with values above 0.90.

The accuracy with which a score value of one instrument can be inferred from the score value of another can be impaired by a systematic lack of agreement between two instruments. The latter was below 0.1 SDs for all conversions. The means of the respective differences ranged from −0.04, 95%-CI: −0.08 – -0.01 (SURV-HD vs. QSC-R23ANX) to 0.06, 95%-CI: 0.02–0.09 (QSC-R23ANX vs. CARQ-3). The 95% confidence intervals that were below zero (i.e., the “line of equality”) had an upper bound of −0.01 and the intervals that were above zero had a lower bound of 0.02, hence the maximum distance from zero was 0.02. Table 2 presents the mean differences (upper triangle) and confidence intervals (lower triangle).

**Table 2:** Mean of the differences between methods of measurement.

	ALL	SURV-HD	SURV-NHO	FOP-Q-SF	FOP-Q-RS	QSC-R23ANX	CARQ-4	CARQ-3	WHITELEY-7
ALL		0.02	0.02	-0.01	−0.02	−0.02	**0.03**	**0.03**	**0.03**
SURV-HD	−0.01 0.05		−0.01	−0.04	**−0.04**	**−0.04**	0.01	0.01	0.01
SURV-NHO	−0.01 0.04	−0.04 0.03		−0.03	**−0.03**	**−0.04**	0.02	0.02	0.01
FOP-Q-SF	−0.03 0.01	−0.07 0.01	−0.06 0.01		−0.01	−0.01	**0.04**	**0.05**	**0.04**
FOP-Q-RS	−0.04 0.01	**−0.08** **-0.01**	**−0.07** **-0.01**	−0.02 0.01		−0.01	**0.05**	**0.05**	**0.05**
QSC-R23ANX	−0.04 0.01	**−0.08** **-0.01**	**−0.07** **-0.01**	−0.04 0.02	−0.03 0.02		**0.05**	**0.06**	**0.05**
CARQ-4	**0.01** **0.05**	−0.02 0.04	−0.02 0.05	**0.01** **0.08**	**0.01** **0.08**	**0.02** **0.09**		0.01	−0.01
CARQ-3	**0.01** **0.06**	−0.02 0.04	−0.02 0.05	**0.01** **0.08**	**0.01** **0.09**	**0.02** **0.09**	−0.01 0.01		−0.01
WHITELEY-7	**0.01** **0.05**	−0.03 0.04	−0.02 0.05	**0.01** **0.08**	**0.01** **0.08**	**0.02** **0.09**	−0.04 0.03	−0.04 0.03	

The upper triangle shows the mean of the differences, the lower triangle shows the respective 95%-confidence intervals of the mean differences (upper value = lower bound, lower value = lower bound). The values in bold face denote the mean differences whose 95%-confidence intervals do not include the value zero.

### 3.4 Common metric

**Figure 2:**
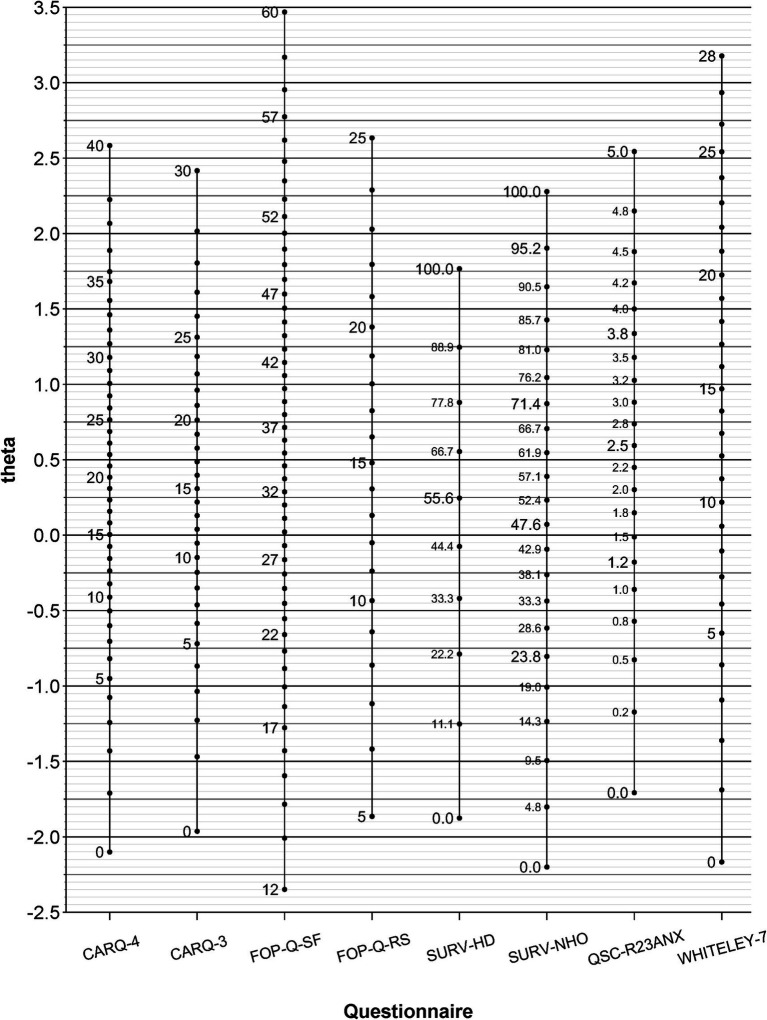
Common metric of health anxiety. CARQ-4, 4-item version of the Concerns About Recurrence Questionnaire; CARQ-3, 3-item version of this questionnaire; FOP-Q-SF, Fear of Progression Questionnaire Short Form; FOP-Q-RS, Fear of Progression Rapid Screener; SURV-HD, subscale Health Distress of the EORTC QLQ-SURV100; SURV-NHO, subscale Negative Health Outlook of this questionnaire; QSC-R23-ANX, anxiety subscale of the Questionnaire on Stress in Cancer Patients–revised version; WHITELEY-7, Whiteley Index; theta, standardized factor score (mean = 0, standard deviation = 1).

Figure 2 presents the main outcome of this study: a common metric to compare the instruments. The left axis shows the standardized factor score (theta) of the general factor. The value zero marks the average level of HA. For each instrument, the data points are labeled with their respective score values in steps of five. For scores that are transformed to a specific value range or that are reported as mean values, the intermediate values are also shown.

Each score value has an average theta value that determines its position regarding the left axis. It indicates a certain level of HA, just as the graduation marks of different thermometers indicate certain temperatures. For example, a score of 28 on the CARQ-4 corresponds to an HA that is about 1 SD above the average level of HA.

Scores that originate from a sample are usually not integer and have decimal places. If a corresponding theta value is to be obtained for such a score from a sample, the theta value can be determined using linear interpolation between score and theta values:


$$\frac{X-X_{\mathrm{lower}}}{X_{\mathrm{upper}}-X_{\mathrm{lower}}}=\frac{\mathrm{theta}\left(X\right)-\mathrm{theta}\left(X_{\mathrm{lower}}\right)}{\mathrm{theta}\left(X_{\mathrm{upper}}\right)-\mathrm{theta}\left(X_{\mathrm{lower}}\right)}$$

Here, X corresponds to the average score value that lies between the score values X_lower_ (below Y) and X_upper_ (above X). The values theta(X_lower_) and theta(Xupper) are the theta values corresponding to the score values neighboring X.

For a mean value in the FOP-Q-SF of 24.93 (Hinz et al., 2015), this results in the following relationship:


$$\frac{24.93-24}{25-24}=\frac{\mathrm{theta}\left(24.93\right)-\left(-0.45\right)}{\left(-0.35\right)-\left(-0.45\right)}$$

If the formula is rearranged to theta(X), the theta value for X is about −0.36.

## 4 Discussion

### 4.1 Common metric

The main objective of this study was to develop a common metric that allows comparisons between scores from different instruments used to measure HA. For example: A study on Danish breast cancer survivors (Ellegaard et al., 2017) reported a mean score of 15.2 in CARQ-4. This value corresponds to a theta value of 0.02. Another study on German breast cancer survivors (Mehnert et al., 2009) reported a mean score of 19.5 in FOP-Q-SF. This value corresponds to a theta value of −0.94. For Chinese breast cancer patients, a study (Ban et al., 2021) reported a mean score of 37.8 in FOP-Q-SF, corresponding to a theta value of 0.78.

By aligning the score values to the level of HA, score values can also be transferred from one instrument to another, that is, cutoff scores for identifying dysfunctional levels of health anxiety can be converted from one questionnaire to another. For example, for the FOP-Q-SF, a cutoff value of 34 or more is considered an indicator of dysfunctional FOP. This score corresponds to a mean HA of around 0.46 above the average level. On the CARQ-3, this theta value corresponds to a score between 16 (with theta 0.40) and 17 (with theta 0.49). This means that patients who report a CARQ-3 score of 17 or more can be assumed to have a similar level of HA as those who report dysfunctional FOP as measured by the FOP-Q-SF.

### 4.2 Measurement precision

In terms of the accuracy of the links in different areas of HA, we can refer to a study on the CARQ (Thewes et al., 2015). The authors report the scores for CARQ-3 and CARQ-4 for Australian and Danish breast cancer patients: For the Australian population the mean scores are 13.0 (CARQ-3) and 16.6 (CARQ-4). Both scores correspond to theta values of around 0.13, i.e., marginally above-average HA. Their lack of agreement (CARQ-4 minus CARQ-3) is below zero and slightly above −0.01 SDs. For the Danish population the mean scores are 5.3 (CARQ-3) and 7.0 (CARQ-4). These scores correspond to theta values of around −0.68 and −0.70 respectively, i.e., to a fairly below-average HA, an area where the reliability of both questionnaires is rather high.

With regard to the reliability of the instruments in the area of average HA (theta range of ±1 SD) CARQ-3, CARQ-4, and FOP-Q-SF showed the highest values, while the lowest values were represented by FOP-Q-RS and SURV-HD. Yet, when looking at the areas outside of average HA, it is noticeable that the instruments lose reliability. This was to be expected because there tend to be fewer respondents in the extreme areas of HA in a sample and the consequently smaller number of cases results in a larger standard error of measurement (SEm). However, the instruments’ reliability can still be differentiated in this area: for moderate HA, the CARQ-3 is the best choice, while in the marginal areas the FOP-Q-SF turns out to be the most reliable instrument.

### 4.3 Dimensionality of health anxiety

The common metric was based on a bifactorial model. As such, the unidimensionality fundamental to a common metric was ensured by introducing a specific factor in addition to the general factor. The specific factor controls for additional work-related influences of individual items that would have systematically distorted the general HA. Overall, the HA can be regarded as a unidimensional construct, as the additional factor had no substantial influence on the parameters relevant for appropriate dimensionality (COH, ECV, PUC).

### 4.4 Limitations

We presented a common metric that also bears potential limitations. These are essentially determined by three elements: the selection of instruments, the sample used, and the model on which the metric is based.

First, the instruments were selected on the assumption that they are widely used in psycho-oncology and that they reflect the specific views of HA. Hence, we focused on a particular set of instruments. The inclusion of additional instruments or the omission of certain instruments may have an impact on the conversions between the instruments used. The extent of the impact depends in particular on which dimensions of HA are ultimately covered by the entire item pool. However, similar metrics could nevertheless be developed for other instruments and linked with this metric using one of the eight instruments included in our study.

Second, the sample is based on a consecutive selection of cancer patients who took part in a rehabilitation program at a large German rehabilitation clinic and who were willing to participate in the study. For patients in this stage of the disease, it can be assumed that the characteristics of HA have stabilized compared to the time of acute treatment. Temporary systematic influencing factors, such as the physical limitations that occurred during acute treatment, have often subsided during this time. The generalizability of the links of this common metric to cancer patients in non-rehabilitative settings or to patients with other chronic diseases is unclear and has not been tested in the area outside of oncology.

In addition, this common metric is not anchored to a reference group of the general population. Hence the results cannot be expressed as T scores referring to the general population.

Third, the metric was developed on a bifactorial IRT-based model to estimate the extent of HA (theta values). A different model would have provided different results and probably at least partially different links between the questionnaires.

A validation of this metric in other clinical settings, as well as in culturally or linguistically different populations could determine or possibly broaden the range of applicability of the metric.

## 5 Conclusion

In summary, the metric presented here enables us to compare results of particular questionnaires with regard to HA and to assess their reliability when used with patients experiencing different levels of HA. This can facilitate the interpretation of test scores and guide the resulting patient care.

## Data Availability

The raw data supporting the conclusions of this article will be made available by the authors, without undue reservation.
