# Supplementary material for: A common metric for questionnaires on health anxiety in cancer patients
Source: Front Psychol. 2024 Dec 3;15:1455121. doi: 10.3389/fpsyg.2024.1455121 (PMC11649417; doi:10.3389/fpsyg.2024.1455121)
Supplement: Supplementary file 1 [file Data_Sheet_1.PDF]

Supplementary Material

1 Supplementary Figures and Tables

Pearson's r	GEN-theta	CARQ-4	CARQ-3	FOP-Q-SF	FOP-Q-RS	SURV-HD	SURV-NHO	QSC-R23-ANX	WHITELEY-7	GEN-score
**CARQ-4**	0.90	0.99	0.98	0.72	0.70	0.77	0.76	0.75	0.71	0.89
**CARQ-3**	0.89	0.97	0.98	0.73	0.70	0.78	0.75	0.74	0.70	0.88
**FOP-Q-SF**	0.92	0.72	0.73	0.99	0.95	0.67	0.77	0.82	0.75	0.91
**FOP-Q-RS**	0.88	0.70	0.71	0.95	0.99	0.65	0.76	0.78	0.72	0.87
**SURV-HD**	0.82	0.76	0.78	0.68	0.66	1.00	0.76	0.67	0.66	0.81
**SURV-NHO**	0.89	0.76	0.75	0.77	0.76	0.76	1.00	0.74	0.75	0.88
**QSC-R23-ANX**	0.88	0.74	0.74	0.82	0.78	0.68	0.74	0.98	0.72	0.87
**WHITELEY-7**	0.86	0.70	0.70	0.75	0.72	0.67	0.76	0.72	0.99	0.85

Supplementary Table 1. Correlations between the measures

Abbreviations: **GEN**: general factor, **CARQ-4**: 4-item version of the Concerns About Recurrence Questionnaire; **CARQ-3**: 3-item version of this questionnaire; **FOP-Q-SF**: Fear of Progression Questionnaire Short Form; **FOP-Q-RS**: Fear of Progression Rapid Screener; **SURV-HD**: subscale Health Distress of the EORTC QLQ-SURV100; **SURV-NHO**: subscale Negative Health Outlook of this questionnaire; **QSC-R23-ANX**: anxiety subscale of the Questionnaire on Stress in Cancer Patients – revised version; **WHITELEY-7**: Whiteley Index; **theta**: standardized factor score (mean = 0, standard deviation = 1); **SEm(theta)**: standard error of measurement.

Notes: The left-outer column "**GEN-theta**" shows the correlations (Pearson's r) between the general factor score and the estimated theta values of the individual measures. The right-outer column "**GEN-score**" shows the correlations between the general factor score and the observed raw scores of the individual measures. The **upper triangle** of the quadratic matrix between these columns shows the correlations *within the raw scores* (values in green color), the **lower triangle** shows the correlations *within the theta values* (values in blue color). The **diagonal** shows the correlations of the respective measure *between theta values and raw scores*.

frontiers

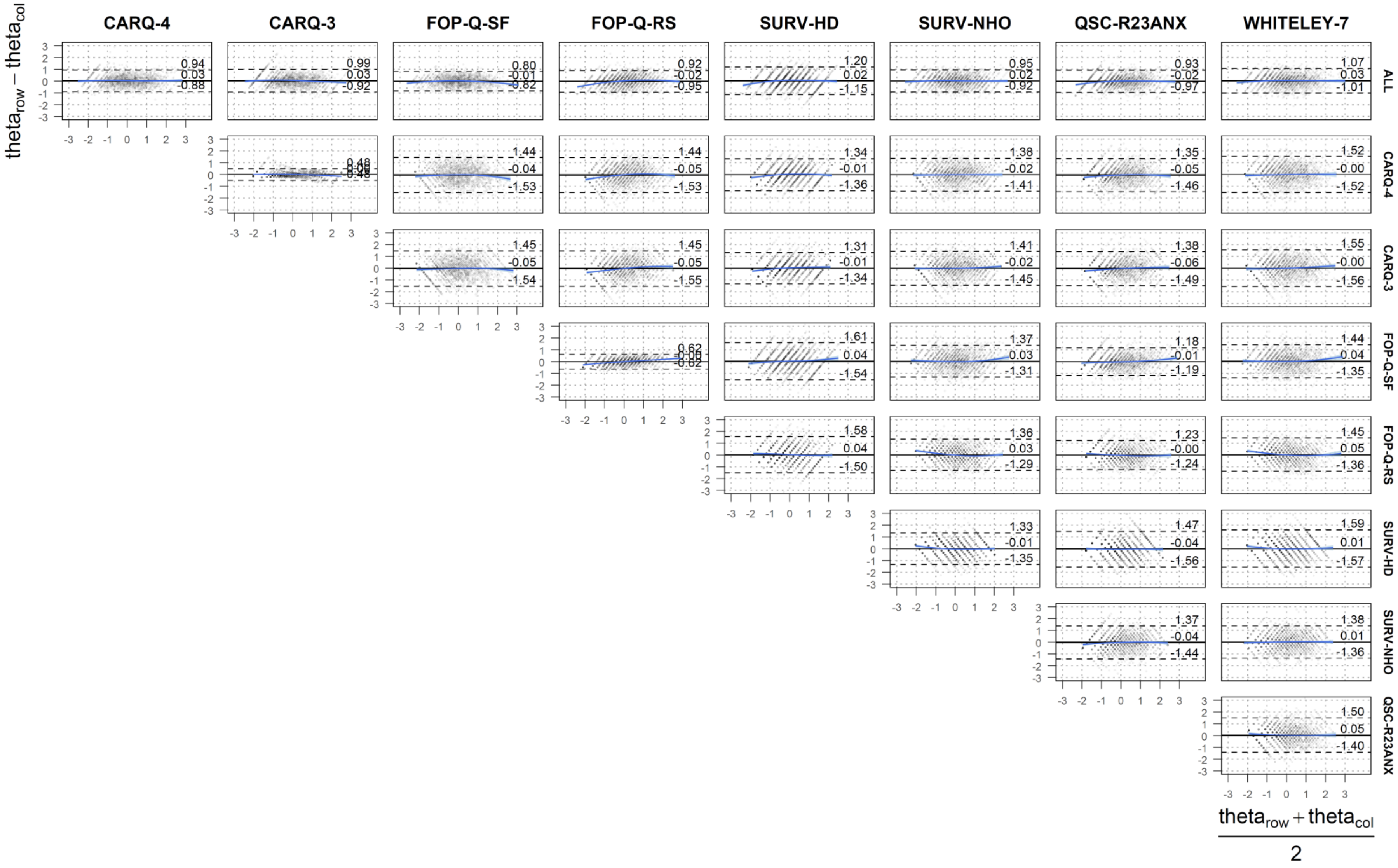

Supplementary Figure S1. Bland-Altman plots.

Abbreviations in supplementary figure 1: **GEN**: general factor, **CARQ-4**: 4-item version of the Concerns About Recurrence Questionnaire; **CARQ-3**: 3-item version of this questionnaire; **FOP-Q-SF**: Fear of Progression Questionnaire Short Form; **FOP-Q-RS**: Fear of Progression Rapid Screener; **SURV-HD**: subscale Health Distress of the EORTC QLQ-SURV100; **SURV-NHO**: subscale Negative Health Outlook of this questionnaire; **QSC-R23-ANX**: anxiety subscale of the Questionnaire on Stress in Cancer Patients – revised version; **WHITELEY-7**: Whiteley Index; **theta**: standardized factor score (mean = 0, standard deviation = 1)

Notes for supplementary figure 1: The plots show the difference between two theta estimates (Y-axis) as a function of the arithmetic mean of the two estimates (X-axis) for each individual data point. A smoothed regression line (blue) represents the course of the mean difference. It should run horizontally near the zero line. To quantify the bias, two additional horizontal lines are shown at a distance of twice the standard deviation from the mean difference. This is where 95% of the differences are located.
